# Traumatic experiences in a lifetime: impact on the connection with others and the role of emotions

**DOI:** 10.1080/07853890.2021.1896197

**Published:** 2021-09-28

**Authors:** Bruno J. Morgado, Gonçalo F. Rodrigues, Marco R. Filipe, Vanessa S. Madureira, Telma C. Almeida

**Affiliations:** a Instituto Universitário Egas Moniz (IUEM), Egas Moniz Cooperativa de Ensino Superior, Caparica, Portugal; b Laboratório de Psicologia Egas Moniz (LabPSI-EM), Centro de Investigação Interdisciplinar Egas Moniz (CiiEM), Egas Moniz Cooperativa de Ensino Superior, Caparica, Portugal

## Abstract

**Introduction:**

Traumatic events in a lifetime have an impact on the connection with others [1] and on emotional regulation in adults [2]. In the present study, we aim to analyse the relationship between traumatic events and the connection with others and to verify the relationship between traumatic events and emotional regulation.

**Materials and methods:**

The study design is descriptive, observational, and cross-sectional. The sample consisted of 63 Portuguese adults divided into two groups according to whether participants had experienced traumatic events (G1: *n =* 28, 44.4%) or not (G2: *n =* 35, 55.6%) with ages between 18 and 61 years old (*M* = 28.78, *SD* = 12.61). The link to the study was disclosed by e-mail and in social networks. Participants answered online to a sociodemographic questionnaire, the Difficulties in Emotion Regulation Scale (DERS) [3], the Adult Attachment Scale-R (AAS-R) [4], and the Childhood Trauma Questionnaire (CTQ) [5]. The study was conducted in accordance with all the ethical principles.

**Results:**

There were significant statistical differences between G1 and G2 on the total scale of the CTQ [*F* (1,61) = 11.510, *p =* .001], and in the subscales Emotional Abuse, Anxiety, and Trusting Others. The total score of the CTQ showed a negative correlation with the Trusting Others (*r*=–0.299, *p*<.05) and a positive correlation with the total score of the DERS (*r* = 0.281, *p*<.05), and with Limited Access to Regulatory Strategies (*r* = 0.337, *p*<.05). There was a positive association between Emotional Abuse and Anxiety (*r* = 0.413, *p*<.05). The Emotional Neglect and the Physical Neglect showed correlations with the DERS, Inability to Engage in Goal-Directed Behaviour, Difficulty to Control Behaviour, and Limited Access to Regulatory Strategies.

**Discussion and conclusions:**

Participants who didn’t experience trauma revealed higher scores of connections with others. Our results also demonstrate that victims of trauma in childhood develop dysfunctional patterns of emotions [6]. This research highlights the negative consequences of child abuse in adults, concerning emotional regulation and connecting with others.
